# Selections

**Published:** 1860-08

**Authors:** 


### Selections.

### REFORMS IN MEDICAL EDUCATION.

THE following remarks upon medical education, are so directly to the point, and occupy the ground so well, that we can not refrain from transferring them to our pages. Every position, and argument here employed, applies as well to the dentist, the student of a specialty, as to the student of general medicine.

Two or three points are worthy of particular consideration, as for instance, shall there be any limit or restriction upon any who seek to enter the profession, especially through the Colleges; this we consider a question of great importance; and one to which, all will admit, sufficient attention has not been given.

Again, occupying the time and attention with that which is more utilitarian in its character, than a long, tedious training in the classics. We have no doubt that many fine minds have been ruined by the system of classic stuffing, which has been so very general. A man who could conjugate a Greek verb, is esteemed a very learned man, though he believed all things to consist of four elements—air, earth, fire and water.

Again, we are pleased to see the attention turned to the investigation of the natural fitness of the aspirant, aside from his literary attainments. We hope all will weigh well the positions taken in the following remarks.—ED.

In discussing this subject we can very naturally divide it into three parts. First—regarding the student who is to be educated; secondly—the science in which he is to be educated; and thirdly—the teacher who is to educate him. In each of these points, our present system of medical education presents deficiencies, demands reforms, and to each apply the resolutions presented and adopted at the late session of the American Medical Association.

Regarding the first point—and to this our remarks to-day will be limited—fundamental questions are involved, and any reform to be brought about, must, from the very nature of the case, be radical. We know this word "radical" is disliked in many quarters, yet we can no better designate the changes which are to be brought about, and which have received the sanction of the American Medical Association, than by this term. They are radical! Why then not say so? They will, if carried out, lead ultimately to a complete and perfect change of the present system; we are performing not more than our duty, when we call this thing by its right name. Besides, in medicine, the term "radical" need not be looked upon as so very offensive. The history of medicine is but the history of the most radical changes; of all sciences, it is the least conservative, and the most progressive. Is it reasonable to expect medical education to become fossilized in old forms, when every day witnesses the evolution of a thousand new facts from their crysalis into independent life? Is it reasonable to expect coming generations to tread the same old beaten track, when the only progress that has ever been made, has been by striking new paths?

But to the point. Shall the admission of young men into the ranks of students of medicine be restricted? This is the practical question. We care not what may be said to the contrary, wise men may show us the printed charters of medical colleges to convict us of an error. We affirm boldly that there is no young man, who has got into his head that he is destined to become a physician, however deficient his elementary knowledge, not to speak of general scientific and classic attainments, but will find some preceptor in good standing to take him into his office, and some college, called regular, in which he can matriculate and from which he can obtain a diploma. If it were necessary at all to argue this point, case upon case might be brought forward in substantiation. We know very well that the great mass of medical students at the present day are possessed of an excellent preparatory education; and we are not at all disposed to join the cry of many who wail and lament, refusing to be comforted, over the scientific status of the rising generation. On the other hand, in reference to the subject of a *classic* education, let it be remembered that in the last fifty years we have actually become five hundred or a thousand years farther removed from the silver age, when we consider the moral effect of what has passed within that time. A man to-day lives *more* in one year than formerly in five. There is no room for much classics in these days. The iron horse has taken the lustre off the famous wooden horse of Troy, and the telegraph wires speak with voice as loud and eloquent as once Cicero or Demosthenes, of the progress of mankind. The indifference towards classic studies, so obvious in our time, has its natural causes. The human mind is limited. To a certain extent the law in physics, that two things can not occupy the same place at the same time, applies to it. Natural sciences have grown up and taken the lead. Something must make room for them. As the individual who devotes all his time and his energy to the study of chemistry, for instance, can not be expected to become a critical classical scholar, or if he has been will soon lose much of his former knowledge; so with the mind of mankind. We might as well lament over the disappearance of the good old traveling coach, as over the gradual downfall and decay of classic studies.

With these remarks, on the one hand knowing that the great mass of the medical students of this day have enjoyed and profited by the opportunities of a good preliminary education, and on the other hand considering the downfall of the old fashioned classic education as natural, and as an evident sign of progress in other directions, we can not be accused of laying too severe a critical measure upon the present system of medical education. Yet we are neither writing a panegyric on the former nor the epitaph of the latter, but a review of facts; and the lamentable truth can not be denied, that the present system of medical education has caused, or, if the term is preferred, has allowed, the admission into our profession of men whose ignorance has cast a dark shadow upon themselves and their brethren, and who, lacking every higher and nobler ambition, have degraded the most liberal of the liberal professions down to a mercenary trade.

The radical change to which we have alluded is, that the indiscriminate admission of young men into the ranks of the profession as students be restricted. Sooner or later, this reform must be carried out. There is, we believe, not *one literary* college in the land, which does not require a preliminary examination. Yet any one who can write his name and the name of his preceptor, though ignorant to the last degree, may enter the temple of our most noble science. It is time that guards should be placed at its very threshold.

But a few words need we say about the manner of restriction. The discriminating power should be vested in the State and District Societies. No student should be allowed to matriculate who can not present certificates of recommendation from the Board of Censors of the District in which he lives. Where such societies do not exist, special censors appointed by the State Society of the respective States in which colleges are located, should be ready at given times to examine candidates for admission. The requirements necessary for admission should be decided by the American Medical Association, and the Boards of Examiners be guided thereby. A distinction between graduates of literary institutions and others should not be made in these examinations for admission. The diploma from a literary college is frequently no more a sign that its holder really possesses literary or classic attainments, than a medical diploma shows that its holder really deserved it. Let there be strict equality—every one on his own merits. Again, without saying any thing against a so-called *classic* education, we would urge the study of *living* languages as important in the preparatory curiculum. If it is a profitable pleasure to crack a joke with Horace, to listen to the powerful invective of Cicero, or to be moved by the sweet pathos of Homeric songs, the study of German and French is not the less profitable and pleasurable to the medical student and the physician. It is a fine thing to read Hippocrates and Celsus in the original, yet it is as pleasing, perhaps more so, to do the same with Virchow and Bernard.

Lastly. A proper restriction, an examination to determine the intellectual and moral capacity of the candidate, how much expectation disappointed and hope blasted would it not present! Alas, how many young men have thoughtlessly entered the open portals of our profession, when but too late they found that they were never made to be physicians! Their money spent, their friends disappointed, their self-reliance gone and hopes blown to the winds, they had the alternative of dragging on a weary, weary existence, practicing mechanically an art which they had not mastered and did not like, cursing it and their fate, or to leave it, regretting the time they had lost in it. Their name is legion. Would they not have blessed their fate in days long after, had some guardsmen stood at those portals and forbidden their entrance!—*Medical and Surgical Reporter.*

### TREATMENT OF NEURALGIA BY SUBCUTANEOUS INJECTION.

BY A. RUPPANER, M. D., BOSTON.

CASE III.—*Neuralgia of the Superior and Inferior Maxillary Nerves, or the second and third divisions of the Trifacial. Injection at different points; relief.*

MRS. ——, of Boston, aged 59 years, married, mother of seven children, of nervous temperament; has suffered from neuralgia for about eight years. The pain is confined to the right side of the head and face, principally to the upper and lower jaw.

What degree of excitability the nerves of sensation of the face may reach, was here most fully illustrated. Would that I were able to describe in adequate terms the indescribable sufferings of my patient; not that I find delight in the recital of a seemingly, too highly-colored tale; no! but to do thereby inadequate justice and homage to the fortitude and resignation with which Mrs. —— has so long borne her suffering. The least breath of air—loud conversation—a sudden noise—the riding in an omnibus over the pavement—even the noise of a passing carriage or other vehicle, over the street—the act of laughing and talking—the taking of fluids, warm or cold, into the mouth—the touching of the gums with the tip of the tongue, would induce a sudden paroxysm of pain, and cause the patient to give vent to her distress in loud screams. At such times the muscles of the upper lip and cheek of the right side are convulsed; and by placing the hand upon the affected part, which is exceedingly painful to the touch, a regular throbbing sensation is distinctly felt, going tick—tick—tick, with perfect regularity, and reminding one very forcibly of the appropriateness of the French name of this malady, "*Tic douloureux.*" The course the pain takes, as it shoots along, is generally regular. Starting from the central and lateral incisor tooth, it shoots upwards to the ala of the nose, thence obliquely upwards and outwards to the infra-orbital foramen, thence to the temple, and finally upwards either to the vertex and again along the suture down to the neck, or from the temple down to the pes anserinus and into the lower jaw.

Is it surprising that the patient's health broke down gradually under so much suffering? For the paroxysms would come on often several times during the day and night—often daily for a week or more, and after a short interval of rest, return to assail her anew. Every thing was tried to give relief, from the medicaments ordered by the most able physicians—which gave temporary relief at least—to the most extolled nostrums of the day.

Sept. 14th.—Examined the patient for the first time, with reference to trying subcutaneous injection. From the direction the shooting pain generally takes, and from the fact of its starting always from the two incisor teeth of the right side, I suspected that much of the trouble was owing to the disordered state of the teeth and gums. Such, upon close examination, did not prove to be the fact exclusively. The gums, however, were in an unhealthy state, and the superior and inferior maxillary bones are, I fear, not in the most healthy condition. Much of the mischief in neuralgia is, no doubt, often owing to decayed teeth; but much harm is also done in indiscriminately extracting teeth, believing them to be the cause of the neuralgia; whilst by removing the tooth, *the nerve, the true seat of the pain*, is by no means reached. Many cases are on record, where no benefit at all was derived from such a procedure; and the present furnishes another illustration of the uselessness of extracting one or more teeth, and the benefit derived from the opposite course when warranted by a correct diagnosis. Several years ago, the subject of the present case had one or more teeth extracted, hoping thereby to get cured of her neuralgia, but in vain. And I believe she was advised by her physician, and her dentist too, not to have them removed. She has still a strong hope, that if her teeth were extracted, there would be an end to her neuralgia. The successful removal of the right molar tooth, of late, seems to have strengthened her in this belief. But the reasons for extracting that tooth, done by my advice, after consultation with Dr. Keep, senior, of this city, and for not extracting the others, will be apparent very soon.

I decided first to try the effect of the valerianate of ammonia on this patient—a preparation, of which I have already spoken. I prescribed, as follows:—℞. Solutionis ammoniæ valerianatis, ʒii.; syrupi simplicis, ℥ ii. M. Cochlear. parv. *pro re natâ.* Also generous diet and pure grape wine. Patient was relieved for the time, but the pain soon returned. I concluded to resort to subcutaneous injection the first time it should return and be severe.

Sept. 15th. — Mrs. —— sent for me, having a terrible access of pain. Pressure revealed the *infra-orbital point* to be the most sensitive; I injected ten drops of the solution at that point. Patient felt a sudden warmth pass over her whole body; (complains of having always cold feet and hands, but particularly of the right side;) five minutes after the operation, or thereabouts, she felt no pain at all, and became drowsy. Left her in that state, lying on the sofa. Œdema at the point of injection inconsiderable; very tender to pressure. Some hours later, she said to me: "What did you inject? That drowsy feeling was splendid; I saw such beautiful visions." Towards afternoon she was seized with nausea, which was shortly followed by vomiting. This continued at intervals until evening, when I prescribed: ℞. Bismuthi subnitratis, ℈i.; infus. gentianæ comp. ℥ iss.; aquæ menth. pip., ℥ ss. M. One teaspoonful every hour till relieved. Vomiting ceased after taking the first dose. Slept well all night, and was free from pain.

16th.—Still free from pain, except directly over the first molar tooth, at the root of which the pain seems to be situated. Patient is very nervous and weak. Prescribed the following: ℞. Infus. gentianæ comp., ℥ iij; extract. valerian., fl ℥ i. M. Two teaspoonfuls three times per day.

17th.—Patient had, last evening, a severe paroxysm of pain in the upper maxillary bone, caused by sudden excitement and much conversation. It subsided after about an hour, under the use of the valerianate of ammonia.

20th.—Was sent for. Patient was very comfortable yesterday, but to-day suffers much from pain in the superior maxillary bone, just at the root of the first molar tooth. Has also pain in the infra-maxillary bone. Injected five drops at the *mental point*, being the most painful point, and about five drops more, close by the right ala of the nose, in a line with the margin of the same. Pain subsided in about ten minutes, and patient felt quite comfortable, with the exception that there was some burning sensation from the puncture made with the instrument. In about ten minutes more a general, comfortable warmth was diffused over the body, and she again passed into a half drowsy state.

21st.—Reports no pain. Comfortable all the rest of yesterday, during the night, and this morning at the hour of my visit. Feels very much debilitated.

26th.—Was requested to see my patient. She reports herself as having been mostly free from pain and more comfortable than ever before, although the weather was very stormy, which had usually affected her very unfavorably. Complains of some pain in the lower jaw, not where I had previously injected, but at the *auriculo-temporal point,* and also at the root of the first molar, as usual. Injected five drops at the *auriculo-temporal point,* and five more near the *ala of the nose.* Patient was at once relieved from pain, and felt easier. I must not omit to state that the patient had mental trouble last week, which may be regarded as the exciting cause of these last paroxysms.

Oct. 1st.—Quite free from pain, with the exception of some slight twinges over the first molar tooth. All the suffering seems to be confined to that place. Patient still takes infusion of gentian and valerian. Tried iron and quinine, but neither agrees with her. Appetite excellent. Pulse 82.

2d.—Complains still of pain over the same tooth as yesterday. Very nervous and excitable about the least thing that is said. Injected again four drops near the ala of the nose. Patient was relieved of pain, but felt very sleepy.

3d.—Reports no pain, but feels much prostrated.

4th and 5th.—Patient exercised both days quite violently. Was exposed to sharp winds. Had, each evening, a paroxysm of most excruciating pain, all starting from the molar tooth. Pain lasts about two hours—from 7 to 9 P. M. Was sent for; when I arrived, somewhat late, pain had subsided. All the pain, which is of a pulling, tearing character, is confined to the tooth. Complains of no pain anywhere else.

6th, 9½ A. M.—Was sent for. Patient had another attack in the same region as on the two previous evenings. Feels very feeble; pulse 72. Injected, directly, five drops over the molar, followed soon by relief. Is sensibly affected by the injection; compares it to a crawling sensation all over the body. Begins to sleep.

7th.—Free from pain. Slept well. Feels weak and prostrated, but not so much as yesterday. Continues her tonic and wine.

16th.—Was called to the patient, who has repeated paroxysms, situated, as before, over the molar. The least touch or motion of the lip produces a paroxysm, which lasts about a minute. Pain does not spread. Injected again with good results.

18th.—Is free from pain, but very nervous. Pulse 104.

19th.—Had several severe, though short, paroxysms this morning. Is entirely prostrated by pain, and extremely nervous. Any and every thing brings on pain. Suffered so severely in my presence, that I injected eight drops of the solution near the *infra-orbital point*, with immediate good result. Patient insists on having the first molar tooth removed, it being the source of all her trouble. Pulse 98.

1 o'clock, P. M.—Consulted with Dr. Keep, sen., as to the removal of the tooth in question, at the patient's request. Dr. Keep had extracted several teeth, within the last few years, for the patient, with no good effect as far as the neuralgia is concerned. The pain always shifted afterwards. Patient is still free from pain, and under the influence of the injection of this forenoon. The lip can be raised without trouble. Is very nervous; pulse 120, with violent palpitation of the heart. For these reasons the operation on the tooth is postponed till next day, in the hope of getting the patient more calm, and pulse reduced.

20th, 9 A. M.—Met with Dr. Keep at patient's house. Has passed the previous afternoon and night free from pain. Went down stairs to breakfast, and had a paroxysm. Pulse 88. Lip comparatively free from pain. Dr. K. extracted the tooth without trouble. The appearance of the tooth presents nothing abnormal, except that its fang is very rough, almost serrated on one side, and more transparent than usual. Says she feels better. At the evening visit the lady is found to be comfortable, free from pain, but still very nervous.

22d.—No pain. Very weak and nervous. Has little appetite. Pulse 92.

24th.—No pain. Feels stronger. Thinks the vegetable bitters and the wine agree with her.

28th.—Patient is still free from pain. Continues to gain strength.

Nov. 3d.—No pain, and much improved. Can eat without difficulty; sleeps well; has been out in the fresh air almost daily; can ride, &c., without suffering from pain.

11th.—Gives a favorable account to-day since I saw her last. Looks better; has a good appetite, and is in good spirits.

From this date my visits ceased; and patient has continued, as I hear, doing well.

I have reported this case in full, in order to present the effects of often-repeated subcutaneous injection; and to show its power of stopping the pain, at least for a considerable period of time, when a possibility of cure is almost, if not entirely out of the question, thus giving, at least, relief from time to time. Here the tic douloureux was so well marked as to leave no doubt in my mind about the nature of the case; the pain was evidently seated principally in the terminal branches of the superior maxillary nerve, in the mandibulo-labralis and some muscular twigs of the inferior maxillary, and to a slighter extent also in the pes anserinus of the portio-dura. Another fact must also not be overlooked in this case. Patient had always a good appetite, although she was unable to eat on account of the pain caused by the motion of the jaws. Having become much debilitated, a tonic treatment was indicated and vegetable bitters produced the desired effect; whilst iron and quinine could not be borne at all. Much benefit, no doubt, was also derived from the constant use of pure Rhine wine.

CASE IV.—*Neuralgia seated in the right temple; Injection at the Temporo-malar point; Use of the Valerianate of Ammonia; relief.*

Mr. ——, residing in Boston, aged 20, book-keeper by occupation, was attacked, some two weeks ago, with violent pain in the right temple, during the night. Is of the sanguineous temperament, and has always enjoyed good health. Is, however, not robust, but rather delicate. Can assign no cause for the pain. Consulted his physician, who prescribed palliative remedies, in the form of ointment, to be applied externally. Did not derive any benefit therefrom.

Sept. 21st.—Consulted me at my office. Pain has been more or less constant; rather dull and heavy instead of lancinating. His teeth are sound. Upon pressure, I discovered the *temporo-malar point* to be the most painful spot of the affected surface. I advised injection, but he rather objected to it, and expressed his preference for internal remedies. Prescribed the valerianate of ammonia in the usual form, and told him to call on me if he did not get relief till morning.

22d.—Reports having obtained no relief from the use of the medicine. Persuaded him to consent to injection. I injected ten drops of the strong solution at the *temporo-malar point.* About fifteen minutes after the insertion of the narcotic, he complained of giddiness, but declared himself free from pain, which was very violent when he entered my office.

Went to sleep for almost an hour. Had no pain when he left me, but the point of injection was very tender to the touch, and slightly œdematous. Patient took tonics for a considerable period afterwards. Pain has not returned, up to the present time.

CASE V.—*Severe pain in the teeth of the right side of the Upper Jaw, occurring during Pregnancy; Injection of Opiates into the gums; temporary and partial, but not permanent relief.*

The patient was a German woman, aged 34 years, of nervo-sanguineous temperament, mother of two children, and four months advanced in her third pregnancy. When I arrived, she complained of pain in all the teeth of the right side of the upper jaw. Suspecting them to be at fault, I examined them carefully, and found them perfectly sound; but on the left side there were two decayed ones. Here, however, she experienced no pain at all. I determined to try the effect of opiates by injection, as everything else had been tried. By means of a curved needle, I injected into the gum fifteen drops of solution No. 4. In about twenty minutes the patient declared her pain to be somewhat less. This being in the forenoon, I called again towards evening, in order to inject once more, so as to give the patient rest over night, if possible, as she had already lost two nights' sleep. Injected again twenty drops of the same solution. Pain was somewhat relieved after half an hour. Patient felt nausea, and soon began to vomit.

When I called next morning, patient informed me that for about two hours after the injection she felt but little pain, but shortly after that time it returned with more violence than ever, and kept her awake all night.

This case not being adapted to the treatment, and having injected for the sake of experiment rather than in the expectation of giving permanent relief, I desisted from any farther operation. The pain subsided, a few days after, spontaneously.

CASE VI.—*Case of Cervico-Brachial Neuralgia of many years' standing; failure of all other remedies; Injection at the Post-Clavicular Point; relief.*

Mr. ——, German, 46 years of age, married, a carpenter by trade, has suffered for many years from severe lancinating pain in his left arm, which he fractured, at the upper third of the humerus, when 12 years old. The pain is generally most severe after exposure to damp or cold weather, or after a hard day's work. Can generally predict, with considerable certainty, the advent of a paroxysm. Pain is sometimes sharp and lancinating, sometimes it partakes of a dull and heavy character. It shoots along the neck, from whence it starts, downwards, is felt all over the shoulder, and is often most severe at the external angle of the clavicle, at its articulation with the scapula. Pressure revealed that the most tender spot was the *post clavicular point* of Valleix. I inserted my syringe within the angle formed by the clavicle and acromion process, and injected twenty drops of the solution I generally use. Not long after the instrument was withdrawn, the patient felt sleepy and drowsy. No nausea nor vomiting. He remained lying on my sofa for an hour, and when he awoke, declared himself free from pain.

Some weeks after, he called again, the pain having returned with increased violence. I injected, at the same point as before, fifteen drops of my strongest solution. The same phenomena were observed, and the same results followed. Having cautioned my patient in regard to his dress and overexertion, and having prescribed some stimulating anodyne liniment, to be used in case pain should be only slight in future, I sent him home. This happened in March, 1859. From that time to the present he has had, in cold, wet and damp weather, occasional and very slight pains, which he says are not worth noticing when compared with his former suffering. Is now working at his trade, perhaps more assiduously than ever.—*Boston Med. & Surg. Jour.*

---

*Paste of the Chloride of Zinc.*—Dr. Spence recommends, in the London Lancet, the following formula for preparing the paste of chloride of zince, so successfully employed in chancroid affections: "Dissolve fifty grains of prepared chalk in two drachms (by measure) of commercial muriatic acid; dissolve a hundred and fifty grains of sulphate of zinc in ten fluid drachms of boiling water. When required for use, mix the two solutions, and the result will be a paste weighing nearly an ounce, and containing one-sixth of pure chloride of zinc."

*On the Physiological Position of Fibrin.* By LEVIN S. JOYNES, M. D., *Professor of Institutes of Medicine in the Medical College of Virginia.*

WE can hardly do justice to this very able article in the brief extracts our space permits us to make from it, and yet it contains too much of interest to allow us to pass it by unnoticed.

Prof. Joynes gives a very just statement of the theory held by some late physiologists, viz.: "That fibrin, so far from being a peculiarly organizable or plastic material, and the immediate pabulum of the most highly vitalized tissues, is, in reality, an excrementitious compound, not at all available for nutrition, and to be reckoned among those elements which have arisen in the blood from its own decay, or have reverted to it from the waste of the tissues, and are in process of elimination from the system."

Prof. Joynes adheres to the older theory, until recently held by all physiologists, which considers fibrin to be a most important element for the nutrition, formation and repair of the tissues; and sustains his opinion by the following arguments:

1st. "Fibrin is a constituent of the chyle. Evident indications of it are found in the fluid drawn from lacteals of an animal in full digestion, at their very issue from the intestine; but its quantity progressively increases by the transformation of albumen, as the chyle moves along the vessels towards the thoracic duct, and through it into the venous system; and a further increase takes place as the blood passes from the venous to the arterial side of the circulation. We may affirm, therefore, that the proportion of fibrin increases as the products of digestion approach the points where materials are needed for the nutrition of tissue; and we may ask if fibrin be an excrementitious product, why should it appear *in the chyle* directly after its absorption? We can not account for its presence here by the waste of tissue, nor can we reasonably suppose the occurrence of a "retrograde metamorphosis" a destructive change in the products of digestion as soon as they are absorbed.

2d. "Fibrin is nomally found only in the *nutritive fluids* of the economy—the blood, chyle and lymph. It is not a constituent of any *excretion*, as are all those constituents of the blood which are admitted to be excrementitious—such as carbonic acid, urea, uric acid, creatine, etc.

3d. "Fibrin is nature's agent for the *arrest of hemorrhage.* When vessels are divided, the coagulation of the blood is the means by which their occlusion is mainly effected, and the flow permanently arrested. If the blood contained no fibrin, and were therefore not coagulable, hemorrhage, even from the lightest wound, could never be arrested by the efforts of nature. But for the same protective property every separation of a gangrenous part would be attended with bleeding. Effusion of fibrin is also the means by which suppuration is circumscribed, and prevented from assuming that diffuse character which is sometimes so destructive. In these several particulars, fibrin performs offices which are singularly *conservative.* Can we say as much for any of these products of wear and tear which constitutes the true offal of the system? It has been aptly remarked, that the organism *bears an increase of fibrin better than a diminution.* Witness the comparative gravity of sthenic inflammation and the severer grades of typhoid fever. Not so with any organic compound of the excrementitious class. The accumulation of these in the blood is the signal of urgent peril.

4th. "Is it mere fancy that sees in the spontaneous coagulation of fibrin, and the definite position which its particles usually assume in solidifying, the indication of a special tendency to organization? And is it unwarrantable to argue therefrom the possession of a certain degree of *vitality?* All attempts to ascribe this coagulation to the operation of mere chemical or physical influences have failed. It is a change which fibrin always undergoes of its own accord, when not kept *moving in contact* with living parts, whatever be the external condition in other respects. Whenever, in the course of the circulation, the plasma of the blood is effused from the capillaries in the midst of the tissues for their nutrition, the fibrin being now *at rest,* is free to pass into the solid state, and enter into combination with the tissue or tissues of which it is the appropriate food. We have no just ground for affirming that fibrin is the only immediate tissue-forming ingredient of the blood; that albumen, for example, which abounds there, must pass through the form of fibrin before combining with any living structure. The probabilities are all against such an exclusive view. But that fibrin is a *specially and eminently organizable or histogenetic* material, this, I am convinced, is a truth which can not be successfully controverted."—*Va. Med. Jour.—Southern Med. Jour.*

*Union of Leather and Metal.*—The Southern Medical and Surgical Journal quotes from a foreign source the following very effectual method for fastening leather upon metal: "The metal is washed with a hot solution of gelatine, and the leather previously steeped in a hot infusion of gall-nuts, pressed upon the surface and allowed to cool. It then adheres so firmly that it can not be separated without tearing."

### SWALLOWING SEVERAL TEETH ATTACHED TO A PLATE, WITH SAFE PASSAGE THROUGH THE BOWELS.

(From the London Lancet.]

The necessity for attention to the teeth artificially attached to a gold plate, when they become out of order, is of so much importance that I beg to trespass upon your space to relate briefly the particulars of the following interesting case, which were communicated to me by Dr. Julius, of Richmond, Surrey:

Mrs. —— swallowed a gold plate on which were three or four teeth, fixed with clasps at each end of the plate for attachment to the adjoining teeth. One of the clasps had become bent, and formed a sharp point. Some hours afterwards she complained of severe pain in the stomach, which seemed very much distended. Dr. Julius made her lie on her right side, and keep quiet. After the lapse of some hours, the pain suddenly left her, and her expression was, that "it went off with a jerk and a pop." She felt nothing more until about twenty-four hours afterwards, when she suffered severe pain in the caput coli, which persisted for some hours, and then passed off in a similar manner. The next morning Dr. Julius was hurriedly sent for, and on arriving found her in great agony, with a constant desire to pass urine and relieve the bowels. Upon passing a catheter into the rectum, he distinctly felt a hard metallic substance firmly fixed across the bowel, about two inches above the sphincter. He now introduced a three-bladed speculum, and dilated the bowel as much as could be borne, and, with the aid of two pairs of forceps, he brought down the sharp end of the plate, and then extracted it, with very slight injury to the mucous membrane. In a few days she was quite well; and, as Dr. Julius observes, after a good washing, used the same teeth again to assist in recruiting her strength after the painful ordeal she had passed through.

The teeth swallowed were two central and two lateral incisors, with the clasp extending to the first molar. One of them had become displaced, and she neglected to have it adjusted. The consequence was they became loose, and were suddenly swallowed, clasp and all.

My object in sending these particulars is to point out the necessity of persons immediately attending to any imperfection in artificial teeth worn in the mouth, however arranged, for the purpose of preventing such accidents as that just narrated, which might have proved more serious, but fortunately for Mrs. ——, terminated without any serious inconvenience. There are many individuals who will not believe that such a piece of plate with teeth attached as that just described, will pass through the bowels at all.

I am, Sir, yours obediently, Thos. H. Harding.

*Park-square, Regent's-park, April,* 1860.

---

Enlargement of Submaxillary Glands after removal of Cancer of the Lip.—A middle-aged man, with an epithelioma of the lower lip, had it removed by a V-incision by Mr. Erichsen, at University College Hospital, in July, 1859. The wound united, and he left quite recovered from the effects of the operation. There was then no enlargement of the glands anywhere. In March he presented himself with swelling of the glands in the right submaxillary region, no doubt contaminated by the original cancerous disease, although the cicatrix in the lip was healthy. Mr. Erichsen observed to his pupils that as he had lately had experience of some of the evils consequent upon leaving glands in this condition, he determined to remove them in the present instance, more particularly as they did not appear to involve the sheath of the carotid artery, although they were probably close to it. Chloroform was therefore administered on the 7th March by Dr. Andrew, and an incision made parallel to the border of the lower jaw. After much patient dissection, two enlarged glands were removed, together with a small portion of the submaxillary gland itself, and in effecting this, it was necessary to divide the facial artery. This vessel with several smaller ones were tied, and the hemorrhage ceased. Nitric acid was applied to a patch of suspicious tissue, which seemed to be the remains of the envelope of the affected glands. The wound was allowed to heal up by suppuration, and as Mr. Erichson believed the disease was entirely local, the man has thus been placed in as favorable a condition as possible for a complete recovery.—*London Lancet.*

### PRACTICAL HINTS.

BY J. D. WHITE.

PLUGGING teeth over slightly exposed nerves, or where there is only a thin plate of dentine covering them, seems at present, from what we see in the journals and discussions before societies, to be quite a settled practice with many in the profession. For more than twenty years we have regarded the practice as unsound, and for as long a time have condemned it, and exerted all our care to avoid plugging over a slight exposure or where the plate of dentine was not thick enough to sustain the life of that portion of the pulp that it was destined to protect. The *liquor sanguinis* supports the vitality of dentine, and when the greater part of the dentine is supplied over any portion of the pulp by a foreign substance, such as a plug of any metal, a *stagnation* takes place between this plug and the pulp, and the thin portion of dentine becomes morbid from an undue amount of nutritive fluid poured into it, the circulation of which has been arrested by the plug, which may be less permeable than the dentine. If there be a considerable portion of dentine intervening, a pulp may *live* without any signs of trouble to the patient, and the slow and normal process of the receding of the pulp, as age advances, may take place by the filling up of the pulp cavity, and the case may be a success for the life of the patient. But if this plate be *too thin*, and by this too thin we mean that according to the capacity of the pulp and the constitution of the patient to successfully sustain morbid action, the case will fail. The stagnated fluids in this portion of dentine ends in decomposing it, leaving an opening between the plug and the pulp or causing a loss of its vitality; in either case inflammation of the pulp is the consequence, and the plug must be removed, and the case treated as if it had been exposed before plugging. We never get into any trouble from this near approach to a pulp, unless it be where we err in our judgment as to the capacity of the pulp to live with a small amount of its natural protection; but we do not destroy a pulp unless we can decide that there is an actual exposure, even though the success of the case may be doubtful; we prefer to give the patient the advantage of that doubt.

We frequently treat such cases with creosote or tannic acid and sulph. morphia, to reduce the action of the pulp for a time after the tooth is plugged, and to compensate for the irritating influences of the jarring the tooth undergoes by the operation; but we are not always successful under what we regard at the time as favorable circumstances. The function of the pulp is to supply nutrition to the crown of the tooth above the gum, as the anatomical arrangement of the tubuli indicate, and the constituents of the circulating fluids to the crown of a tooth is equal to the wants of that substance, as is true of any other tissue of the body; and how our modern physiologists provoke the pulp to produce more of one constituent than another, seems to us to be too absurd for serious consideration.

We have always been taught and believed that to increase the action of an organ or a tissue, that an excess of the fluids was the result of that increased action instead of the solids. How to approach a pulp of a tooth by *therapeut'c agents*, so as to procure a rapid deposit of dentine or bony matter, and reduce the vascular and nervous tissue of the pulp at the same time, is beyond our comprehension. If the earthy constituents are increased, the animal constituents and fluids are increased also, and, as far as we can judge in treating teeth, where the pulp is irritated or increased in its action, this is true. "*Ubi irritatio ibi fluxus*" is, we believe, sound doctrine. Upon removing a plug for pain in a tooth, when the pulp is living or a recent case of irritation, a small portion of serum will be found between the plug and the pulp, and if it be a case where intense pain has been experienced, it frequently happens that pus and blood will also be found, the accumulation of which will have compressed the pulp so that the orifice of exposure can be entered without touching the pulp, but in a few minutes after the discharge of the pus or serum, the pulp will regain its normal size and fill its cavity, and the pain will cease, which had been caused by the pressure; hence the relief that is obtained by giving "vent," as it is termed.—*Dental Cosmos.*

In the above practical hints we find a mode of practice that has obtained quite extensively in the dental profession, rather unceremoniously condemned. It is always well to give a caution when there is danger, especially to those of small experience.

It strikes us as rather a peculiar method of disposing of any point of practice, to say that "for twenty years we have condemned it, and considered it unsound," and of course have not practiced it, while the profession generally have considered it good, and have introduced it into practice, and with very generally good results.

We have practiced filling cavities in which there was a thin lamina of dentine covering the pulp, for fifteen years, and with a success that warranted a continuance of the practice. Occasionally a pulp will die, where the circumstances are unfavorable; but if we should abandon everything that might have unpleasant occurrences connected with them, we would soon find ourselves standing still in the world.

We know that quite a large number in the profession have been indulging in this "condemned, unsound practice," without the least intimation that they were sinning against correct principles.

In regard to this matter we state what we think is correct practice, based upon experiment, which, when thorough, amounts to demonstration.

In all cases where a tooth is decayed and the pulp is covered by a lamina of dentine, however thin it may be, if there is not actual exposure, we fill. We do not operate alike in all cases; where the constitution is good and the pulp healthy, and but little or no sensitiveness of the dentine, we fill at once with gold. If there is much sensitiveness of the dentine, that should be remedied by the appropriate treatment. If the pulp is in an irritable condition, by its near exposure to the changes of temperature, then such treatment should be employed as will restore it to health; and usually it will be quite sufficient for this to cleanse out the cavity, and introduce a temporary filling of "Hill's stopping" or os-artificial, which should remain from ten days to two months, according to the indications. During this time, the parts being protected from the influence of irritants, will have attained a healthy condition, and the pulp will have a better protection, and in nineteen cases in twenty, the tooth may be filled with gold with *success.* Of course, rather more than usual care is requisite in such cases. Where the pulp is slightly exposed, and not too much diseased, the same course may be pursued, and with skillful management, with about the same results. In the majority of cases, with favorable circumstances, a small orifice of exposure, when well protected from foreign influences, will be closed up with bony deposit from the pulp in from ten days to two months, and then it is just as safe to fill as though the pulp had never been exposed. But if it still remains open, and the pulp exposed, then at once make all as healthy as possible, and place over the orifice of exposure a protection of a proper non-conducting material, then introduce a good filling of gold, and all will be well.

We employ other methods of treating such cases, a description of which we have given heretofore.

The Doctor thinks it too absurd for a moment's consideration, that the pulp of a tooth should throw out osseous material more rapidly when a tooth is diseased than when it is healthy, or that it can do anything out of the ordinary course to meet an exigency. How is it in other parts that sustain an injury, as when a bone is broken? Does nature do nothing to meet an exigency? have the parts been provoked to produce more of one constituent than another? is it too absurd for consideration? Nothing more is done in the case of the tooth than the fractured bone. In both cases there is an extra elaboration of bone material, which we think every one can understand, without being very absurd or ridiculous.

